# Colorectal Cancer and Colitis Diagnosis Using Fourier Transform Infrared Spectroscopy and an Improved K-Nearest-Neighbour Classifier

**DOI:** 10.3390/s17122739

**Published:** 2017-11-27

**Authors:** Qingbo Li, Can Hao, Xue Kang, Jialin Zhang, Xuejun Sun, Wenbo Wang, Haishan Zeng

**Affiliations:** 1 School of Instrumentation Science and Opto-Electronics Engineering, Precision Opto-Mechatronics Technology Key Laboratory of Education Ministry, Beihang University, Xueyuan Road No. 37, Haidian District, Beijing 100191, China; zy1517312@buaa.edu.cn (C.H.); kuangxianghua@buaa.edu.cn (X.K.); zhangjl2014@buaa.edu.cn (J.Z.); 2 Department of General Surgery, First Hospital of Xi’an Jiaotong University, Xi’an 710061, China; sunxy@xjtu.edu.cn; 3 Cancer Imaging Unit—Integrative Oncology Department, BC Cancer Agency Research Centre, Vancouver, BC V5Z 1L3, Canada; wenbo.wang.ca@gmail.com (W.W.); hzeng@bccrc.ca (H.Z.)

**Keywords:** Fourier transform infrared spectroscopy (FTIR), colorectal cancer, pattern recognition, entropy weight local-hyperplane k-nearest-neighbor (EWHK)

## Abstract

Combining Fourier transform infrared spectroscopy (FTIR) with endoscopy, it is expected that noninvasive, rapid detection of colorectal cancer can be performed in vivo in the future. In this study, Fourier transform infrared spectra were collected from 88 endoscopic biopsy colorectal tissue samples (41 colitis and 47 cancers). A new method, viz., entropy weight local-hyperplane k-nearest-neighbor (EWHK), which is an improved version of K-local hyperplane distance nearest-neighbor (HKNN), is proposed for tissue classification. In order to avoid limiting high dimensions and small values of the nearest neighbor, the new EWHK method calculates feature weights based on information entropy. The average results of the random classification showed that the EWHK classifier for differentiating cancer from colitis samples produced a sensitivity of 81.38% and a specificity of 92.69%.

## 1. Introduction

Every year, the number of cancer-caused deaths rises [1]. Among all types of cancer, colorectal cancer is the third most common cause of cancer death worldwide, with an annual incidence of approximately one million cases and 600,000 deaths. The high mortality rate is partially attributed to the fact that established clinical procedures lack reliability and sensitivity for finding cancer at early stages [2,3]. Thus, the importance of early diagnosis in preventing and treating cancer mandates development of an accurate, fast, convenient, and inexpensive diagnostic tool for early detection [4].

Substantial modifications in cancer cells at the molecular level occur prior to morphological changes could be observed in tissues. Therefore, molecular spectroscopes are promising tools to detect cancer-related chemical changes at an early stage [1]. In particular, Fourier transform infrared spectroscopy (FTIR), a popular tool in modern analytical chemistry labs, provides rich information about the bio-molecules that act as building blocks in tissues and cells [5,6,7,8]. Existing clinical diagnosis requires taking biopsy via endoscope, which causes pain and requires lengthy pathological exams. In addition, surgical resection can lead to taking biopsies from non-cancerous tissues due to a number of factors, and there is always a possibility that malignant cells could go into blood stream during such invasive procedure. Therefore, being able to diagnose colorectal cancer in vivo or ex vivo could largely overcome the limitations of existing procedures, providing accurate and rapid determination of proper operative treatment. Combining an attenuated total reflectance (ATR) fiber probe-coupled FTIR spectrometer with an endoscope, a simple, rapid, and noninvasive method to detect human cancer tissues directly with minimal sample preparation may achieve results comparable to the gene expression-based method [9,10,11,12,13]. The current work was carried out on ex vivo tissues using an ATR-FTIR probe.

In recent years, the use of FTIR to diagnose various cancers, such as lung, breast, gastric, liver, and colorectal cancer, has been reported [14,15,16,17,18,19,20,21,22,23]. Chemometric methods, such as support vector machine (SVM) [21], K-nearest neighbor classifier (KNN) [22], and K-local hyperplane distance nearest neighbor (HKNN), enable efficient information extraction and classification model calibration [24,25]. These afore mentioned reports indicate that FTIR spectroscopy along with an effective chemometric classifier could be a useful tool for screening a variety of human tumors. Until now, few studies have been developed for diagnosis and discrimination of colorectal cancers and colitis using FTIR spectroscopy [26]. Most research efforts focus on enabling a high-accuracy and high-sensitivity algorithm for cancer diagnosis. In this study, we combine preprocessing techniques and a novel classification method for analyzing FT-IR spectral data and achieve high accuracy in diagnosing colorectal cancer tissues.

## 2. Materials and Methods

### 2.1. Tissue Specimens

All colorectal cancer and colitis tissues were provided by the Medical Division of the First Hospital of Xi’an Jiaotong University, China. Informed consent was obtained from each patient prior to the study, and clinical diagnosis was confirmed by histopathology. A total of 88 tissue samples from 42 female and 46 male patients, were obtained. The average age was 53.7 years old with the oldest being 76 years and the youngest age being 21 years. One fresh endoscopic biopsy of 1–3 mm in diameter was obtained from each patient. According to the pathological exam results, the samples consisted of 41 cases of colitis and 47 cases of cancer.

### 2.2. Instrumentation and FTIR Data Collection

A WQF-500 FTIR spectrometer linked with a modified attenuated total reflectance (ATR) fiber probe (Beijing No. 2 optical instrument factory, Beijing, China) was used to acquire spectra. The FTIR spectrometer was equipped with a liquid-nitrogen-cooled mercury cadmium telluride (MCT) detector. Specimens were frozen and transported to the laboratory. Before experiment, frozen specimens were thawed at room temperature for approximately 3–5 min. Then, a background spectrum was acquired first. The ATR probe was placed at a 90° angle on the tissue specimen surface for spectrum acquisition. To achieve an acceptable signal-to-noise ratio at a resolution of 4 cm^−1^, 32 scans were recorded with wavenumbers ranging from 1000 cm^−1^ to 4000 cm^−1^. The procedure took approximately 1–2 min. After sample spectra were recorded, samples were stored in liquid nitrogen and sent for the histological examination as reference for spectral analysis.

### 2.3. Spectra Preprocessing Method

Two preprocessing methods, viz., smoothing and standard normal variate (SNV) [27,28,29], were performed on the FTIR spectra. First, the Savitsky–Golay algorithm with a window width of 5 points was applied to each spectrum to reduce random noise in the data. Then, all available spectra were normalized by the SNV method to remove multiplication interference, slope variation, and scatter effects generated by particles of the sample.

For spectrum $x_{ij}$ of sample i at wavenumber j, SNV standardization is defined as follows:

(1) $$x_{ij,SNV}=\frac{x_{ij}-{\bar{x}}_{i}}{\sqrt{\sum_{j=1}^{n}{\left(x_{ij}-{\bar{x}}_{i}\right)}^{2}}}{\left(n-1\right)}^{1/2}$$

where ${\bar{x}}_{i}$ denotes the average spectrum of sample i, n denotes the number of wavelengths, and (n−1) denotes the degree of freedom.

### 2.4. Entropy Weight Local-Hyperplane K-Nearest Neighbor Method

A novel classification method called entropy weight local-hyperplane k-nearest neighbor (EWHK) is proposed for discrimination between colorectal cancer and colitis. For the EWHK method, which is an upgrade of K-local hyperplane distance nearest neighbor (HKNN) algorithm [24,25], feature weights of training sets based on the information entropy are objectively considered to measure the importance of each single feature and to avoid the bias in high dimensions and the limit in small values of the nearest neighbor. On the other hand, HKNN treats every variable as an equally relevant component for classification. Therefore, the class labels of unknown samples are calculated according to the feature weights, the Euclidean distance, and the local hyperplane.

Suppose that training set $X={\left(x_{1},\cdots ,x_{m}\right)}^{T}$ consists of m training instances with L classes. Each training instance consists of n input features $x_{i}={\left(x_{i1},\cdots ,x_{in}\right)}^{T}$ with known class label $y_{i}=c$, for i=1,⋯,m and c=1,⋯,L. The class label of a query with input vector $q={\left(q_{1},\cdots ,q_{n}\right)}^{T}$. The three stages in the proposed method were as follows: prototype selection, local hyperplane construction, and query classification.

Firstly, the feature weight is estimated objectively based on the concept of information entropy to figure out the entropy weight according to the variance of every variable. Low information entropy resulted in high feature weight, which corresponds to a feature with better class separation capability. The entropy weight $w_{j}$ is calculated according to the following formula:

(2) $$\begin{array}{l} z_{ij}=\frac{x_{ij}}{\sum_{i=1}^{m}x_{ij}},\beta =\frac{1}{\ln(m)}\\ H_{j}=-\beta \sum_{i=1}^{m}z_{ij}\ln(z_{ij})\\ w_{j}=\frac{1-H_{j}}{n-\sum_{j=1}^{n}H_{j}},\;\forall j=1,\cdots ,n \end{array}$$

where $z_{ij}$ denotes the normalized jth component of sample i in the training set; β denotes the regularization parameter; $H_{j}$ denotes the information entropy of the jth feature of the sample. Hence, new weighted Euclidean distance metric D between $x_{i}$ and q is defined as follows:

(3) $$D(x_{i},q)=\sqrt{\sum_{j=1}^{n}w_{j}{\left(x_{ij}-q_{j}\right)}^{2}}.$$

Then, a local hyperplane of class c is constructed for the given query q according to the distance metric D and the number k of nearest neighbors of class c. Formally, the formula is as follows:

(4) $$\begin{array}{l} LH_{c}(q)=\left\{s|s=\sum_{i=1}^{k}\alpha_{i}V_{.i}+m_{c}\right\}\\ m_{c}=\frac{1}{k}\sum_{i=1}^{k}p_{ci}\\ V_{.i}=p_{ci}-m_{c}\\ \alpha ={\left(\alpha_{1},\cdots ,\alpha_{k}\right)}^{T} \end{array}$$

where $p_{i}$ is the ith nearest neighbor of class c; α is solved by minimizing the distance between q and $LH_{c}(q)$ using regularization. Thus, the calculated minimum distance is as follows:

(5) $$\begin{array}{l} J_{c}(q)=\min_{\alpha }\sum_{j=1}^{n}w_{j}{\left(V_{j.}\alpha +m_{cj}-q_{j}\right)}^{2}+\lambda \alpha^{T}\alpha \\ \;=\min_{\alpha }{\left(s-q\right)}^{T}W(s-q)+\lambda \alpha^{T}\alpha \\ W=diag(w_{1},\cdots ,w_{n}) \end{array}$$

where λ is the regularization parameter. $J_{c}(q)$ is minimized, and the equation $\left(U^{T}V+\lambda I_{k})\alpha =U^{T}(q-m_{c}\right)$, where $U^{T}=V^{T}W$ is used to calculate α, is derived. Finally, the class of the query q is assigned as follows: $class(q)=\arg\;\min_{c}\;J_{c}(q)$.

## 3. Results and Discussion

### 3.1. Preprocessing of FTIR Spectra

In the process of measurement, the obtained spectra contain not only useful information regarding the molecular structure and the components of the measured samples, but also the noises, such as the high-frequency random noise, the baseline drift, and the stray light. This additional noise needs to be eliminated; otherwise, they will affect the discrimination result.

**Figure 1:**
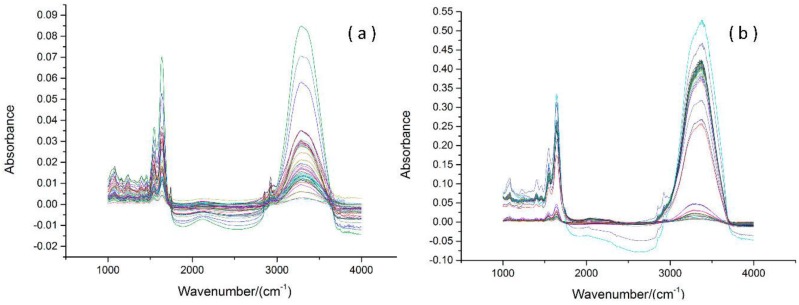

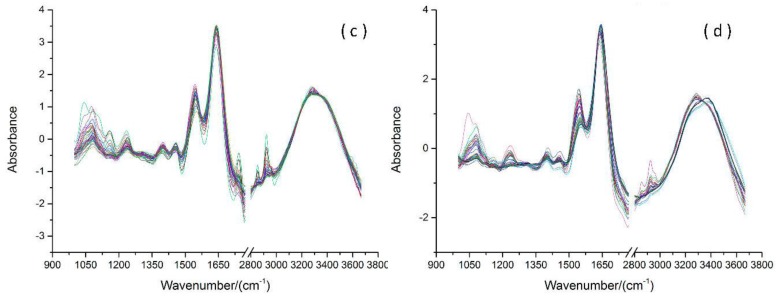
The Fourier transform infrared spectra (FTIR) of colitis and cancerous tissues. (**a**) Original spectra of colitis tissues; (**b**) original spectra of cancerous tissues; (**c**) preprocessed spectra of colitis tissues using smoothing and standard normal variate (SNV); (**d**) preprocessed spectra of cancerous tissues using smoothing and SNV.

**Figure 2:**
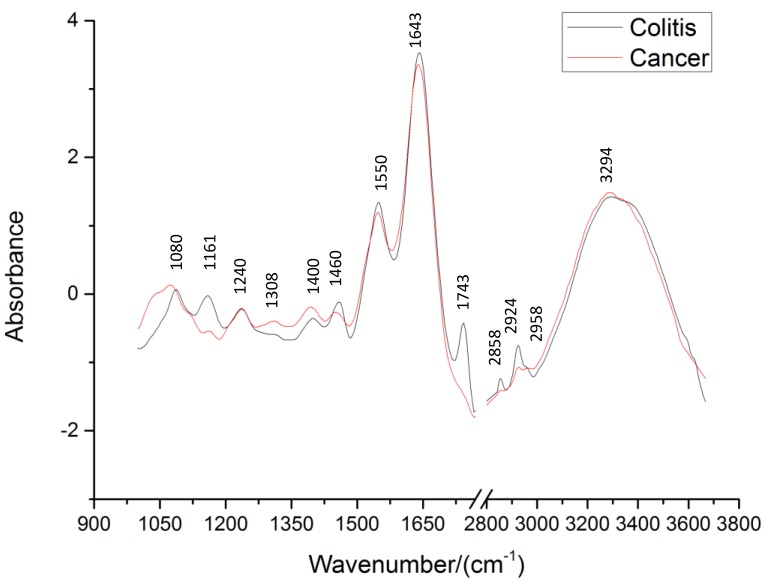
The typical FTIR spectra of colorectal biopsies after preprocessing.

Prior to classification analysis, data preprocessing is necessary to improve performance of the classification model. Savitzky–Golay (SG) smoothing reduces random noise, and SNV was applied to remove unwanted background variances to some extent. There is no bio-molecules absorbance peak in the 1800–2800 cm^−1^ region, the majority of peaks are in the 1000–1800 cm^−^^1^ region and in the 2800–3800 cm^−^^1^ region. Thus, the SNV method was separately used from 1000 to 1800 cm^−1^ and from 2800 to 3800 cm^−1^.The FTIR spectra of colitis and cancerous tissues before and after preprocessing are shown in Figure 1. After performing background correction and normalization, useful information of all spectra (such as at the wavenumber near 1743 cm^−1^, 2858cm^−1^, and 2924 cm^−1^) were marked as shown in Figure 2. The quality of FTIR spectra was greatly improved after data preprocessing.

### 3.2. Analysis of FTIR Spectra

A total of 88 spectra were obtained by FTIR spectroscopy within the spectral region between 1000 and 4000 cm^−1^. Because there were no bio-molecule absorbance peaks in the 1800–2800 cm^−^^1^ region, the SNV method was separately used from 1000 to 1800 cm^−^^1^ and from 2800 to 3800 cm^−^^1^ after smoothing.

Figure 2 shows the spectra of colitis and colorectal cancer biopsies after preprocessing, where the band assignments of major absorption in the FTIR spectra of colorectal tissue are marked. The major peaks are similar for the spectra of colitis and colorectal cancer. However, the differences including peak shape and relative intensity can be observed. These results are reasonable because significant changes occurred in both the structure and composition of the main bio-molecules, which constitute the cell such as DNA, water, protein, and lipids, between cancerous and colitis tissues.

As is shown in Figure 2, the spectral profile of cancerous tissues indicates the presence of fewer lipids and more proteins compared with colitis tissues. The peak intensity of the C=O band assigning to the lipids (near 1743 cm^−1^) and the peak intensity of C-H stretching vibration bands relating to the lipids (near 2958 cm^−1^, 2924 cm^−1^, and 2858 cm^−1^) decrease and even disappear in the spectra of malignant tissues, making it essential to consume fat in the malignant tissue to meet the nutritional and energy requirements in carcinoma development. The spectral profile of cancerous tissues indicates the presence of proteins at wavenumbers ~1643 cm^−1^ and ~1550cm^−1^, which belong to amide I band and amide II band of the protein, respectively. The relative intensity near $I_{1550}/I_{1643}$ decreases more for the spectra of cancerous tissues than for those in colitis biopsies because of the changes in the proportion of proteins during tumor formation. The intensity of the ~1460 cm^−1^ peak is weaker than that of the ~1400 cm^−1^ peak in the spectra of the cancerous samples, while the peak at ~1460 cm^−1^ is stronger than or equal to that of ~1400 cm^−1^ in the spectra of colitis samples. Cancerous tissue contains greater amounts of nucleic acids, collagen, and certain amino acids compared to the colitis ones. In colitis tissues, the peak at ~1240 cm^−1^ is weaker, and the band near 1310 cm^−1^ becomes weak and sometimes disappears. The absorption peak ~1080 cm^−1^ assigned to nucleic acid is obviously weaker in the spectra of colitis samples than that in the spectra of cancerous tissues. The peak at ~1160 cm^−1^ assigned to carbohydrate decreases noticeably in the spectra of the cancerous samples. Thus, the characteristics mentioned above between cancerous and colitis tissues provide the basis for spectroscopic diagnosis.

Specific assignments of individual peaks can be found in Table 1.

### 3.3. Classification Analysis

After spectra preprocessing, 88 spectra data (41 colitis and 47 cancers) were analyzed to identify their class labels. The total 88 spectra were divided into two data sets. The 44 FTIR spectra (21 colitis and 23 cancers) after preprocessing were randomly selected as the training set. The other 44 FTIR spectra (20 colitis and 24 cancers) after preprocessing were randomly selected as the test set. Both EWHK and traditional classification models were built by the training set and validated by the test set. Traditional classification models include SVM and HKNN in this paper. These procedures were repeated five times. The five predicted results were averaged. Table 2 shows the classification results of colorectal tissues with entropy weight local-hyperplane k-nearest neighbor (EWHK). Table 3 shows the average of the five results using the three different methods.

The experiment results are summarized in Table 2 and Table 3. The classification results of colorectal tissue samples with EWHK (Table 2) showed that, among the 88 cases of colorectal tissue samples, only three colitis samples and nine cancer samples are misclassified. In Table 3, EWHK achieved a high accuracy, viz., 85.91%. In addition, other statistics results of detection of colorectal biopsies by FTIR spectroscopy with EWHK and traditional classification models are shown in Table 3. For colorectal cancer diagnosis with EWHK, sensitivity is 81.38%, specificity is 92.69%, predictive value of a positive test is 92.68%, and the predictive value of a negative test is 80.85%. In comparison, statistical analysis results with HKNN were worse than those with EWHK in diagnosing colorectal cancer tissues, achieving 66.46% sensitivity and 79.77% accuracy. The SVM works worse than HKNN. SVM can perform well with large-scale data. However, the choices of the parameters for the kernel are complex and unstable. The HKNN works well only for small values of the nearest-neighbor. However, the accuracy decreases as values of the nearest-neighbor increase. The FTIR spectra can be classified accurately with EWHK because it considers the influence of feature weight according to the information entropy of every variable. In conclusion, the results indicate that the EWHK has better capability in identifying colorectal cancer from colitis.

## 4. Conclusions

This study shows that it is feasible to classify colitis and cancers using FTIR spectroscopy and chemometrics. FTIR fiber-optic ATR spectroscopy is a powerful tool to detect changes at the molecular level and can rapidly capture small changes in molecular compositions and structures. Therefore, it has the potential to be further developed into noninvasive, in vivo, and real-time detection tools of cancerous tissues before a surgical operation is required. Data pre-processing such as smoothing and SNV greatly improved the signal-to-noise ratio for the FTIR spectra of colorectal tissues, and the EWHK classifier achieved a classification accuracy of 85.91%. The reason that EWHK performs well is because feature weights are calculated according to the information entropy of every variable. The proposed preprocessing and classification method using FTIR spectroscopy is effective and practical for in vivo colorectal cancer or other malignant tissue diagnosis and will be pursued in future studies.

## Figures and Tables

**Table 1:** Peak positions and assignments of FTIR bands in colon tissues.

Peak Positions (cm^−1^)	Major Assignment
1080	Stretching vibration(DNA, RNA)
1160	Carbohydrate
1240	Asymmetric stretching vibration(RNA)
1310	C-H deformation vibration & Amide III (protein)
1550	amide II (protein)
1643	amide I (protein)
1743	C=O stretching vibration(lipids)
2858, 2924, 2958	C-H stretching vibration (lipids)
3300	N-H stretching vibration( protein), O-H stretching vibration(water)

**Table 2:** The average of predict results of colorectal tissues with entropy weight local-hyperplane k-nearest neighbor (EWHK).

Histologic Examination	The Predicted Results of Fourier Transform Infrared Spectroscopy (FTIR) Spectra	
	Cancer	Colitis
Cancer	38	9
Colitis	3	38

**Table 3:** Comparison of the average of statistical analysis results with several classification models.

Method	Sensitivity (%)	Specificity (%)	PPV ^1^ (%)	NPV ^2^ (%)	Accuracy (%)
EWHK	81.38	92.69	92.68	80.85	85.91
HKNN	66.46	96.19	95.25	70.43	79.77
SVM	72.46	68.70	70.30	71.87	70.45

Note: ^1^ Positive predictive value; ^2^ Negative predictive value.
